# From Classics to Nano-Excipients and Biopolymers: Pulmonary Drug Delivery Formulations

**DOI:** 10.3390/pharmaceutics18010108

**Published:** 2026-01-14

**Authors:** Maria Nikou, Maria Chountoulesi, Stergios Pispas, Natassa Pippa

**Affiliations:** 1 Section of Pharmaceutical Technology, Department of Pharmacy, School of Health Sciences, National and Kapodistrian University of Athens, Panepistimioupolis Zografou, 15771 Athens, Greecemchountoules@pharm.uoa.gr (M.C.); 2 Theoretical and Physical Chemistry Institute, National Hellenic Research Foundation, 48 Vassileos Constantinou Avenue, 11635 Athens, Greece; pispas@eie.gr

**Keywords:** dry powder inhalers (DPIs), excipients in dry powder inhalers, technology of DPIs, DPI formulations, pulmonary delivery

## Abstract

In this article, a systematic review and analysis of the present literature is conducted, regarding the excipients present in dry powder inhaler formulations. Until now, there has been no list of excipients recorded, specifically for DPIs, with the number of approved excipients for pulmonary delivery being restricted, despite their choice as a pivotal step for the formulating process. Understanding the DPI formulations, physicochemical characteristics, efficiency, and release profiles, demonstrated in detail here, could contribute to their application in future studies and be a useful research tool in the choice of excipients in the field of inhalation technology and specifically DPIs.

## 1. Introduction

Dry powder inhalers (DPIs) are gaining attention for local and systemic pulmonary drug delivery for the treatment and diagnosis of various diseases [1]. More than 40 different types of DPIs are on the market, and drug repurposing is becoming a reality. Since they are complicated systems and are considered drug–device combination products (European Medicines Agency-EMA), they require deep understanding of the complex formulation–device–patient interplay.

The first dry powder inhalation device, Spinhaler^®^, was developed in the late 1960s to early 1970s for sodium cromoglycate powder administration [2]. Since 2000, DPI patents started growing with the approval of blockbuster drugs and inhibitors, playing an important role in DPI active ingredient patents with 1039 patent documents covering inhibitors. Apart from PDE-4 inhibitors and interleukin inhibitors, which have been widely discussed in DPI applications, other small-molecule inhibitors have recently been investigated [3]. DPIs are described as propellant-free inhalers that deliver dry particles of aerosol to the lower respiratory tract, containing three functional parts: a powder–drug formulation, a dosing system (single- or multi-unit dose), and a physical powder dispersion mechanism. Some also contain a drug metering system. Additionally, features regarding patient feedback are incorporated onto some DPI devices, such as visual or auditory indicators, confirming the correctness of the inhalation performed [4]. Currently, widely established techniques include top–down jet milling followed by blending for carrier-based formulations and bottom–up spray drying and new emerging technologies, such as spray freeze drying and thin film freezing [5]. DPIs can be categorized into single/unit-dose, and multi-dose (unit or reservoir) integral devices. Other classifications include passive or active devices, according to powder aerosolization mechanisms, and low-, medium-, and high-intrinsic resistance. Additionally, DPIs are updated through three generations: first-generation, breath-actuated single-unit dose devices; multi-dose reservoir-type second-generation devices; and third-generation devices that employ active inhalation technology to disperse and deliver drugs through applied energy [6].

Lung-targeted delivery presents advantages, such as a large alveolar surface area > 100 m^2^, rich blood supply, low enzymatic activity, high permeability of the thin peripheral epithelial layer (0.2–0.7 μm), and the ability to surpass first-pass metabolism. Thus, rapid onset of action, with no gastrointestinal discomfort or mechanical damage, and better patient compliance, due to the non-invasive approach for treatment and diagnosis, is achieved. By local treatment, increased drug deposition in target tissue and reduced toxicity related to high systemic exposure presents possibly better results [7].

DPIs present a reduced need for coordination between actuation and inhalation, enhancing drug delivery, with no spacer required. The ideal inhaler is summarized as portable, easy-to-use, inexpensive, and able to maintain drug stability, with DPIs leading in that direction. Additionally, they enable the delivery of high drug payloads to the lungs.

Despite these advantages, DPI technology is challenging since the optimum aerodynamic particle size is between 1 and 5 μm, with many factors influencing engineering. Particles smaller than 1 μm are eliminated by coughing or exhaling, and even smaller particles with a median aerodynamic diameter (MMAD) < 0.5 µm are driven by Brownian diffusion in the alveolar region [8] or exhaled. Particle size above 5 μm results in deposits by inertial impaction on walls, which remain in the upper respiratory tract and are mucociliary cleared. Lungs are extremely humid, so particle hygroscopicity should be considered with more exposed, solid formulations, resulting in aggregation. However, with the technique of Excipient Enhanced Growth (EEG), high humidity and hygroscopicity could be exploited, using a hygroscopic excipient such as mannitol and a dispersion enhancer, like L-leucine. Also, low solubility and sustained release present a challenge for the rate and extent of absorption. The complex airway nature and heterogenous properties of lung areas are demanding. Critical for the dissolution and absorption of drug particles, drug–mucus interaction and lung defense mechanisms limit drug delivery, as alveolar macrophages are sensitive to particles ranging 0.5–5 μm in size [9]. Usually, the small amount of an API in a single dose, especially in carrier-based formulations, limits reproducibility in each actuation [5,10]. Most inhaled products are administrated multiple times/day and have a short half-life, therefore impacting patient compliance and treatment efficiency. Also, as with all inhalers, errors in use still happen (13–15%) [4].

This review article aims to present an overview of the most common excipients, explained, categorized, and summarized as mentioned below, based on selected scientific results. Herein, an in-depth examination of the excipients used in DPIs is conducted, as this field is still under development, and further research into potential excipients in DPI formulations to evolve this technology and existing knowledge is essential for future formulations. To the best of the authors’ knowledge, this is the first report in the literature of an in-depth analysis of the role of the excipients used for the design and development of pulmonary drug delivery formulations, taking into account their added value for the fast clinical translation of DPIs. Additionally, the authors attempt to find a classification roadmap based on a statistical breakdown of the excipients in order to help formulation scientists find the best candidate(s) for each active pharmaceutical ingredient and further investigate it/them during preformulation and formulation studies.

## 2. Methods

The research methodology in this work included searches in the electronic databases “PubMed” and “Google Scholar” for articles published within the last 5 years, from 2019 to 2024. The search terms were the English words “Dry Powder Inhalers” and “DPIs formulation” along with specific keywords, such as “Excipients in DPIs” and “Technology of DPIs”. The systematic review and analysis included approved, investigational, and emerging excipients in terms of effectiveness, efficacy, functionality, and added value for pulmonary drug delivery.

From the articles studied, excipients in DPIs formulations could be divided into four main categories:

1. The most dominant in pharmaceutical forms;
2. Biopolymers;
3. Nano-excipients;
4. Others/non-categorized.

The results presented suggest that the dominant and most frequent type of excipients used in DPIs are solid pharmaceutical forms, followed by biopolymers, nano-excipients, and others.

## 3. Excipients in DPIs

In this systematic analysis, the major functional categories of excipients are covered.

- Carriers/bulking agents (lactose, mannitol);
- Force control agents/dispersibility enhancers (magnesium stearate, leucine);
- Stabilizers (lactose, trehalose, and mannitol);
- Lubricants;
- Moisture protectants/hygroscopicity modifiers.

The physicochemical properties influence DPI performance and for this reason are considered critical quality attributes (CQAs). Namely, particle size/size distribution as quantified by the aerodynamic diameter is responsible for lung disposition. The morphological characteristics, shape, and surface roughness are responsible for the flow and adhesion properties. Brittleness is the driving force for the de-aggregation and de-agglomeration phenomena during inhalation. Last but not least, surface energy governs the inter-particulate interactions and consequently powder dispersibility and aerosolization effectiveness. Special discussion is addressed in each example obtained from the literature on the critical quality attributes of DPIs, especially about dose uniformity, aerosolization, fine particle fraction (FPF), flow properties, and physicochemical characteristics.

### 3.1. The Most Dominant Excipients in DPIs: Functions and Applications

Regarding the most frequent type of excipients in DPIs, most of them are sugars or amino acids.

The most common excipient used as a carrier in pulmonary delivery is in solid form, and it is α-lactose mono hydrate [11,12,13]. A-lactose is the alpha anomer of a disaccharide composed of galactose and glucose and is primarily used as a diluent and flowability enhancer to improve the dosing reproducibility of micronized API particles. A-lactose is stable, biodegradable, biocompatible, safe, and cost-effective [14]. Although FDA has approved the use of lactose as a carrier for DPI formulations, lactose is not suitable for lactose-intolerant patients and for the delivery of some drugs (e.g., Formoterol, peptide, and protein drugs) since it is a reducing sugar. Accordingly, several studies have investigated other alternative carriers such as mannitol, trehalose, erythritol, sorbitol, xylitol, and maltitol [15,16].

Starting with lactose, binary mixtures of model drugs (salbutamol, aceclofenac) and lactose monohydrate were prepared separately at varied drug loading doses (1–33 wt.%), and a physicochemical evaluation was performed to study the influence of surface interactions of drug–excipients. Mixtures of aceclofenac (AC)- lactose monohydrate- (LM) and salbutamol sulphate (SS)-LM were prepared using a Turbula mixer. As seen using a Field Emission Scanning Electron Microscope (FESEM), with 10–14% drug loading, particles tend to agglomerate with increasing concentrations of cohesive drug particles. At higher drug loading doses, drug particles were attached to the excipient surface homogeneously. Insights from several techniques were compared with XPS results, indicating the formation of condensed or addition compounds. Thus, the presence of polar protic groups in the drug can result in interactions with reducing sugar, such as lactose monohydrate, leading to unwanted condensed and combined compounds. Therefore, the formation influences the mixing and bulk properties of mixtures, resulting in increased %RSD and cohesion. Using a coating layer for the protection of the protic group and surface modification of the excipient with Magnesium stearate (MgSt), aerosil R972 (model nanoparticle) eliminates inter-particle interactions and crystal-level changes, and interactions can be prevented and reduced, improving the drug content uniformity and bulk flow properties of the mixtures [17].

Another formulation of a powder particle blend of Terbutaline Sulphate (TBS) and CapsuLac 60 (mass ratio 1:9) was prepared, and the blending vial was tumbled using a Turbula shaker-mixer. During the preformulation, adhesive carbon tabs were pre-mounted onto aluminum stubs and then sputter-coated with gold to achieve a thickness of around 30 nm to reduce electrostatic charging. The mixture also included tableting-grade α-lactose monohydrate powders, inhalation-grade α-lactose monohydrate (Lactohale 100 (sieved) and Lactohale 200 (milled), and Tablettose 70). It should be underlined that 3D technology is an important tool for examining new and potentially expensive formulations and early drug development phases and analyzing powder blends within capsules or DPIs, and it could even be utilized as a process-line tool for quality assurance. Also, with the non-destructive nature of XCT, invaluable three-dimensional insight into the structure of pharmaceutical powders can be provided [18].

In another study, two fine excipient materials, micronized lactose particles and silica microspheres coated with dimethylpolysiloxane, as DPIs, with a coarse lactose carrier (50–200 μm) and Fluticasone Propionate (inhalable (D < 5 µm)) were investigated to study their influence on aerodynamic performance and identify critical attributes of fine excipient materials. The drug material was prepared via nano spray drying, while inhalation formulations were prepared with a sandwich mixing technique. The results show that silica microspheres were more narrowly distributed than the micronized lactose particles, more flowable, and more tightly packed. Both materials showed fine excipient fractions and FEF10.0, greater than 90% *v*/*v*. Particle shape was different depending on the fine excipient, with quasi-spherical particles for the spray-dried drug. Also, bulk differences in the excipient blends were present, with micronized lactose; bulk density decreased as compressibility increased with increasing fine material concentration, while permeability decreased. At 2.5% *w*/*w*, both excipient materials similarly improved drug dispersibility, while at higher concentrations lactose material was more beneficial. Additionally, the ratio of cohesive to adhesive interactions was greater in the lactose formulations. Regarding aerosolization properties, the only difference was in the evolution of the initial burst. So, physical characteristics could strongly influence the capacities of the fine excipient particles, which improved drug dispersibility at low concentrations by filling carrier surface macropores and forming agglomerates and/or enforcing fluidization at higher concentrations [19].

From recent studies, it was observed that MgSt could decrease powder retention in an inhaler, resulting in an improvement of emissions, potentially for high-dose inhalation powders via co-jet-milling. However, according to Li and Wu, there are several things to consider when including magnesium stearate in a formulation, such as compatibility with APIs and the compatibility of common impurities in MgSt batches with APIs. Further research is needed to confirm if the low water solubility of MgSt and the possible interaction with lung surfactants may not be a limiting factor for the use of higher doses of MgSt. However, the amount of magnesium stearate approved is relatively low, with maximum content per unit dose for inhalation enlisted in the inactive ingredient list of the FDA, as only 80 mg, with additional concerns about toxicity in higher doses. An investigation into the role of dispersion enhancers on the aerosol performance of DPI formulations of Tratinterol hydrochloride (TH) was performed, using magnesium stearate (MgSt), micronized MgSt (MgSt-M), and fine lactose (FL). The particle size of TH-M and MgSt-M was smaller than TH and MgSt as received, with all enhancers in an inhalable size range, less than 3 µm. More fine particles and agglomerates observed in the DPI formulations consisted of 10% of FL. Also, from NGI, it was shown that the addition of MgSt or FL significantly decreased the emitted dose, and MgSt-M was more effective than MgSt-R in improving the fine particle fraction (FPF) [20].

Additionally, the effect of lactose fine addition in 0.5% MgSt, containing DPI formulations, and their mixing on powder and aerodynamic properties was studied, using Fluticasone Propionate (FP) as a model drug. Seven DPI formulations were prepared, consisting of 0.5% MgSt with different amounts (1–10%) of lactose fines (F1–F5), various mixing orders of 0.5% MgSt and 5% lactose fines (F3, F6), and mixtures containing only 5% lactose fines (F7) to investigate the potential synergistic effect of lactose fines and MgSt in improving FPF. The addition of lactose fines (1–10%) in 0.5% MgSt improved flowability and enhanced adhesion. In drugs with strong drug–lactose adhesion, the combination of MgSt and lactose fines can both enhance the FPF of the DPIs and reduce the amounts of lactose fines required for similar FPF values, thus avoiding potential safety concerns caused by too many fines. So, the addition of lactose fines can synergize with MgSt to enhance aerosolization performance under the mixing order of carrier–MgSt–lactose fines–drug with a linear relationship between powder rheological parameters and FPF. Regarding carrier-based DPIs, the strength of drug–carrier adhesion (i.e., whether the drug can be detached from the carrier smoothly) is a key factor in determining the aerosolization performance [21].

### 3.2. Amino Acids as Multifunctional Excipients in DPI

Amino acids are typically used in DPI formulations to improve aerosolization and powder dispersibility due to their ability to change the physicochemical properties of dry powders. Small-molecule amino acids were studied as stabilizers and filling agents in DPIs. L-leucine presented dispersibility and long-term stability enhancement characteristics. Amino acids, such as L-leu and trileucine, are used to reduce particle cohesiveness. L-leucine is the most investigated amino acid in inhalation formulations, with results showing changes in the surface composition and potentially the morphology (e.g., corrugated) of the resulting particles. L-leucine has low solubility and low diffusivity, resulting in a high Peclet number (Pe) during spray drying and migration to the surface of the droplet, forming a crystalline shell early [22].

Leucine was used to develop co-spray dried powders for the inhalation of leucine and lactose to examine the potential interaction of the two excipients. According to DSC analysis, a new endothermic peak at 177 °C, and the disappearance of pure lactose and leucine endothermic peaks, could be a sign of incompatibility. FT-IR data demonstrated that the spectra in both co spray-dried powders were comparable, and interaction occurred after spray-drying. From SEM and XRD, the amorphous state of the powder with a smooth surface and spherical shape is confirmed. The interaction that might occur could alter the physicochemical characteristics of the product, decreasing efficiency and changing stability and appearance. It should also be mentioned that during DPI manufacturing in higher temperatures (above 130 °C) and humidity, alterations might be observed regarding protein activity and the distinctive brown hue powder, especially because of the Maillard reaction. Further studies are needed to evaluate this interaction [23].

In another study, Excipient Enhanced Growth (EEG) powder formulations for inhalation were developed, containing Tobramycin as the model drug, D-Mannitol as a hygroscopic excipient, and Poloxamer and L-leucine as the dispersion enhancer. The EEG technique employs micro-sized particles of a hygroscopic excipient, such as mannitol, which increase in size following inhalation to minimize upper respiratory tract deposition and maximize targeted deposition in the lungs. The main DPI design was created using Autodesk Inventor and was exported to be 3D-printed. The production of the formulations was performed using the spray-drying process. Lower L-leucine concentrations showed better aerosol performance with MMAD around 2 μm and FPF < 5 µm around 80%. Also, it is possible that enrichment of an L-leucine hydrophobic layer on the surface of the spray dried particles can act as a protection against water uptake. Finally, further studies are needed to conclude if the control of spray-drying conditions will lead to an improvement of the solid stability of the Tobramycin EEG formulation and a change in the aerosol performance of the formulation [24].

A proof-of-concept study was performed to investigate the spray-drying effect of Tout, atomization flow rate (Rotatom), and feed flow rate (Ffeed) on powder properties and on the enzyme activity and protein stability of trehalose—leucine spray-dried powder with Cu-Zn-superoxide dismutase (Cu-Zn-SOD). The spray-drying technique (SD) was used to prepare dry powder for inhalation. It was observed that the particle size and aerodynamic performance of the SD powders was mainly influenced by Rotatom, the enzyme activity was affected by Rotatom and Ffeed, and the stability was influenced by outlet Temperature (Tout), which presents a critical parameter. The inner particle morphology was also affected by Tout. Also, it is noticeable that a higher Tout presented faster drying kinetics and larger leucine surface enrichment of the shell. Additionally, a correlation between particle size distribution value Dv50, MMAD, and FPFED is observed, with lower Dv50 resulting in lower MMAD and higher FPFED. The information from this study shows that higher values of Tout, Rotatom, and Ffeed can potentially be employed for a successful spray-dried Cu-Zn-embedded trehalose—a leucine inhalable powder formulation [25].

A combination powder using two model drugs, ceftazidime and roflumilast, was prepared to target infection and inflammation with a two-step approach. The first step included the identification of an amino acid and its concentration (% *w*/*w*) for the best aerosolization enhancement of ceftazidime, using different ratios of leucine and tryptophan in combination. For the second step, leucine was kept constant at 25% *w*/*w*, the percent with the best FPF (75%), while roflumilast (5–20% *w*/*w*) was incorporated into the formulation. As a result, ceftazidime content seemed to decrease to compensate for the introduction of roflumilast into the formulation; leucine crystallized, and the FPF decreased to 55%. So, it was noticeable that the inclusion of a second drug into drug amino acid amorphous matrix particles can affect its solid-state dynamics and aerosol performance, changing the surface and matrix nature of the particles. Also, the spray-drying process did not alter the anti-bacterial property of ceftazidime, nor the anti-inflammatory effect of roflumilast. Further studies are needed to better understand the interactions among all the investigated drugs and excipients to achieve the desired performance and formulation characteristics [26].

Drug repurposing of ebselen, as an amino-acid-incorporated, spray-dried DPI formulation, incorporating amino acids such as leucine, methionine, and tryptophan for the treatment of respiratory tract infections (RTIs) was attempted. Amino-acid-incorporated spray-dried powders showed a higher process yield, with tryptophan-containing dry powder showing the highest. Particle size ranged between 1.8 and 2.8 μm, with median diameters of the size distribution measuring between 1.6 and 2.7 μm, suitable for inhalation. Regarding particle morphology, differences between the amino acids were observed. From the aerosolization properties, the highest average ED and FPF were achieved by leucine-containing dry powder. It was shown that amino acids may decrease the surface energy of dry powder, thus reducing cohesiveness, which eventually could enhance aerosolization, and this is highly dependent on drug structure. As for cell viability, use of leucine in the inhalable formulation can lower the potential cytotoxicity. Furthermore, from antibacterial and antibiofilm activity studies, amino-acid-containing formulations did not alter antimicrobial activity, with none of the compounds showing biofilm inhibitory activity below the determined MIC. Further studies of such formulations should be performed to better understand the use of amino acids in DPIs with individual and/or combinations of antimicrobials for both upper and lower RTI treatment [27].

Spray-dried inhalable protein powders with proteinX and varying L-leucine concentrations were developed to select two formulations (5% *w*/*w* and 10% *w*/*w* leucine) based on the best aerodynamic characteristics for further evaluation of leucine’s influence on aerosolization stability. A leucine crust was only visible for leucine contents of 10 and 20% *w*/*w* and did not cover the whole droplet, while when leucine content was doubled from 10% to 20%, protein aggregates increased, and an increase in leucine concentration resulted in lower water content, given that the amino acid was more hydrophobic than the protein itself. Also, during single droplet drying, the less leucine the better, and the evaporation rate was generally lower the higher the leucine content. It is shown from the study that less leucine results in more physically stable formulations, and, thus, further investigations are needed to develop a low-dose, protein X-Leu, inhalable final product, which would be aerosolization-stable and have an improved FPF, as well as be biocompatible and effective [28].

The crucial role of leucine could also be proven from the study of two different active pharmaceutical ingredient (API) solutions, consisting of both a biologic and a small molecule, simultaneously atomized through separate nozzles into a single-spray dryer and collected by a single cyclone to produce a uniform mixture of two different active particles in a single-unit operation. The mixture contained bevacizumab with erlotinib, cisplatin, or paclitaxel in a dry powder inhaler formulation, chosen for potential local treatment of lung cancer. For the preparation, solutions for the spray drying of paclitaxel, cisplatin, or erlotinib were prepared by adding API and excipient solids to the solvent and stirring until dissolved, and bevacizumab solution preparation was performed via dialysis buffer exchange. Water content, quantified by the Karl Fischer test, is an important parameter. When it is too high, recrystallization of amorphous domains, particularly trehalose, can cause failure, and if it is near-zero, static dominates the powder, resulting in poor release. From SEM, both ingredients exhibited comparable particle size distributions and were successfully and independently atomized. Also, MMAD was between 1.5 and 3 μm, and the sizes of both types of particles within the simul-spray formulations had aerodynamic diameters that were appropriate for the pulmonary target. The presence of crystalline L-leucine was critical in presenting good aerosol properties. Additionally, the produced powders preserved anti-VEGF bioactivity and could be a combination treatment to locally treat lung cancer, achieve patient compliance, limit doses, and reduce toxic exposure. This process could eliminate additional blending operations on poor flowing inhalation powders and potentially shorten development timelines. For future studies, one formulation could be combined with smaller particle sizes to target the alveolar region of the lung, while a second could be sized to target the conducting airways [29].

In another study, Salvianolic acid B (Sal B) DPI was prepared via spray drying, using Sal B as a model drug and L-leucine (LL) as an excipient, with modern preparation evaluation methods to assess the quality of Sal B-DPI. Most particles of each formulation were smaller than 5 μm, suitable for DPI. Regarding particle morphology, surface roughness of SD Sal B particles increased with the amount of LL, with most particles being spherical or nearly spherical, with a corrugated surface or hollow with a smooth surface (as found by SEM). It was observed that during long-term storage, the formulation gradually changed to a crystalline form; thus, monitoring storage conditions is important. Also, LL enhances the anti-hygroscopicity of DPI to a certain extent, thereby improving the stability of the drug. Additionally, when LL increased, the in vitro deposition properties of DPI improved. The formulation of 80SalB20LL was selected as the best ratio for stability and subsequent tests, showing stability for 35 days. The formulation was proven to cause no irritation to the lungs, overcoming the shortcomings of poor oral absorption and low bioavailability of Sal B, increasing the drug concentration in the lesion site, and improving the therapeutic effect of IPF, therefore being a possible option for clinical treatment [30].

Furthermore, the effect of L-leucine concentration, as an aerosolization enhancer, on the aerosolization properties and bioactivity of inhaled spray-dried viral-vectored vaccines was examined. The mean geometric particle size of spray-dried samples ranged from 5.7 to 9.0 µm, with a present increase when L-leucine concentration increased. From SEM images it was also shown that, at higher concentrations, leucine appears to have a dominating influence on the surface of particles, as evidenced by its crystalline nature. The formation of a dry crust at the droplet surface is observed, followed by partial collapse as internal solvent evaporation continues. Due to the crystalline nature of L-leucine, the crystalline shell helps prevent droplet collapse and promotes the formation of hollow, well-defined, low-density particles with wrinkled or corrugated surfaces, causing shrinkage of this outer shell and the appearance of wrinkling, related to leucine enrichment on the surface. Regarding aerosolization influence, only at the highest leucine concentration used (50% LL-MDsample) was the FPF/AD higher, so powders with >40% FPF and a low aerodynamic diameter of 4.2 µm could be achieved, relative to the control mannitol/dextran formulation. Thus, the anticipated deposition of particles in the central and peripheral airways was similar for all formulations except 50% LL-MD. Additionally, it was observed that the most common mechanism for the degradation and loss of virus activity was molecular aggregation in the spray-drying solutions, with increased leucine reducing in vitro viral activity. In conclusion, leucine has both a positive and a negative impact on viral vaccines, with increased viral dosage loading to balance the loss of the effective dose. This is a potential field of research for future studies [31].

Effectively prepared by the emulsion-solvent evaporation method, Triamcinolone acetonide (TAA)-loaded poly (lactide-co-glycolic acid) (PLGA) dry powder inhaler (DPI) formulations were developed using the spray-drying process, with mannitol and leucine as carriers. From SEM images, particles were spherical with smooth surfaces, and the particle size was determined by laser diffraction between 2.7 and 3.1 µm. All formulations were negatively charged, and it was noticed that an increase in TAA increased the negative zeta potential. Also, the entrapment efficiency for all microparticle formulations was greater than 41%, with the concentration of PLGA in the oil phase as a critical factor, regarding the entrapment efficiency of PLGA microparticles containing TAA. As for the release profile, a biphasic release with an initial burst, followed by slower release, was observed. In conclusion, this formulation presented suitable aerodynamic characteristics for lung administration. An improvement in the management of asthma might be possible with prolonged drug delivery to the lungs, increasing the efficacy of the therapy and decreasing the administrated dose with a useful inhaler dosage form [32].

It was discovered that leucine could be combined with biopolymers such as phytoglycogen (PyG), as excipients, for eFPF enhancement during the study of levofloxacin DPI formulations. Optimization of spray-drying parameters with the use of a face-centered central composite design to amplify deep lung delivery was also performed. Regarding the particle shape from SEM images, needle-like or other irregular shapes were observed, but after the addition of PyG into the formulation, the spray-dried particles (SDPs) presented deflated ball shapes. By optimizing the selected spray-drying parameters of inlet temperature, feed flow rate, and gas flow rate, successful alveolar delivery was observed from the simulated throat that was utilized. Also, it is important to add that the most critical parameter was the gas flow rate, owing to its significant contribution (*p* < 0.05) to droplet size, regarding the atomization force, which is coherent in most articles of engineering. In addition, the extra-fine particle fraction of optimized formulations of LVFX was >5%. So, the potential of the design space, also tested in rifampicin and still considered as a work in progress, might be useful in preparing efficient inhaler formulations with general validity, since most responses were at the 95% prediction interval. Additionally, the results might be a possible tool for alveolar-targeting dry powder inhalers consisting of leucine and large polysaccharide excipients [33].

In another study, Meloxicam inhalable “nano-in-micro” DPIs were developed with additives such as poly (vinyl alcohol) and leucine, aimed at the respiratory zone, with micro-composites and a higher drug concentration with a nano-active ingredient. The formulation was prepared by combining wet milling (pre-nanosuspension, PVA, MX) and spray-drying (microsized powders, LEU). The particle size was within the inhalable range (3–4 μm), was spherical (SEM), and was monodispersed in all cases—essential parameters for accurate dosing. Excipients also showed reduced density, resulting in deeper reaching of the airways. Release of half of the drug within the first 5 min and most within an hour was observed in samples with nano API, and this was related to nanosizing effects, higher specific surface area, and amorphization. An increase in polarity presented with PVA also prevented aggregation and helped the release of MX in the simulated lung medium. As for LEU, as the concentration increased, there was an increase in the API diameter, as can be observed in Table 1. Also, leucine reduced the cohesion between the particles, resulting in larger amounts of MX liberated from the powder and increased deposition in deep airways. From in silico studies, higher values were present in the acinar region than in the bronchial region, proving delivery in lower parts. The samples showed good aerodynamic measurements for an effective local treatment for lung diseases, which should be tested in vivo soon, including stability testing [34].

### 3.3. Mannitol: An Alternative Option

Mannitol seems to be the best alternative since it is animal-free and extraordinarily stable [35]. It is a non-reducing sugar, contrary to lactose—a reducing sugar capable of causing a Maillard reaction with amines. Thus, non-reducing sugars can be employed for peptide- and protein-based drugs [36].

**Figure 1:**
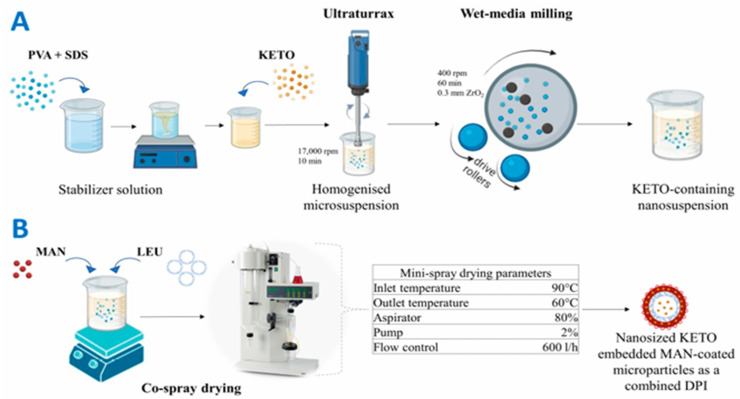
Preparation methods using particle engineering techniques: (**A**) preparation of the pre-dispersion (ketoprofen-containing nanosuspension); (**B**) preparation of nanosized ketoprofen-embedded mannitol-coated microparticles as combined dry powder for inhalation. KETO: ketoprofen; MAN: mannitol; LEU: leucine. Adapted from [37].

Mannitol has been approved as add-on maintenance therapy to improve the pulmonary function of adult patients with cystic fibrosis. For instance, a DPI comprising nanosized ketoprofen (KETO)-embedded mannitol-coated microparticles was developed for the treatment of pulmonary inflammations. A ketoprofen-containing nanosuspension was prepared, as observed in Figure 1, by a wet media milling process combined with a prior homogenization step and KETO nanosized with PVA and SDS (pre-dispersion) by ultraturrax and the wet-milling method, and then co-spray dried with leucine as the dispersity enhancer and mannitol as the coating and muco-active agent. The particle size of the ketoprofen-containing nanosuspension was ~230 nm. SEM images of the spray-dried powder displayed wrinkled, coated, nearly spherical particles with a final size of ~2 µm (nano-in-micro), which is optimal for pulmonary delivery. From the dissolution study, a release of ~80% of the drug in 5 min was observed, because of the mannitol, which is highly wet, and the nanosized KETO. The developed formulation might be ideal for local inhalation and treatment of lung inflammations, with low cytotoxicity and no observed decrease in mucus viscosity. Also, a careful adaption of mannitol concentrations is important, due to the potential decrease in efficient loading of this excipient in higher concentrations. In the in silico study, all samples led to a higher deposition when breath-holding time was increased to 10 s. Further evaluation, including in vivo studies to optimize the formulation, is needed [37].

Wet media milling is an attractive particle engineering approach which enables a simple scale-up in terms of industrial nanosuspension manufacturing.

Additionally, large porous microparticles of mannitol–leucine (MLAs) were developed using the spray-drying technique, investigating the variation in ammonium bicarbonate and leucine concentrations on the particle morphology and physicochemical properties. Co-processing with leucine, a force control agent selected to improve particle dispersibility and aerosolization for the DPI formulation, was applied, with the hydrophobic nature of leucine serving to enhance the physicochemical stability of the material, repressing the moisture-inducing deterioration effect. Also, mannitol seemed to exist only in the most stable polymorphs. From the FESEM study, the morphology of MALs displayed a spherical hollow shape with a corrugated surface (when leucine up to 2.5% was used), improving powder dispersibility with enhanced aerodynamic lift and reduced settling velocity. The formulation was suitable for inhalation, with no observed interactions nor permeability to the lower respiratory tract, as particles were less than 5 μm and tapped density below 0.4 g/mL. In summary, the optimal ratio was MAL with 10% leucine with suitable characteristics for respiratory tract delivery, and further research should be performed on the ability of MAL as a carrier for deep lung areas [38].

In another study, highly respirable dry-powder vaccine particles were produced with a three-fold repeated peptide epitope from minor capsid protein L2, displayed on Pyrococcus furiosus thioredoxin as the antigen. Amphiphilic endotoxin derivative glucopyranosyl lipid A (GLA) was used as a built-in immune adjuvant, a lubricant, and a coating agent for particle de-aggregation and respirability. Mannitol was selected as the best bulking agent for supplementation of the feed solution and both an indicator of antigen emission from the device and its good distribution within the different stages of the impactor. From SEM images, more irregular shapes were observed for higher (2.00% *w*/*w*) antigen concentration. Additionally, the GLA-containing powders displayed the desired flow properties, thermostability, and low moisture absorption. The dry-powder vaccine, always handled under non-refrigerated conditions, had no physicochemical or aerodynamic differences from its first development and maintained its activity for 5 months in room temperature, important for potential clinical use. Thus, the developed formulation might be a future candidate for needle-free, easy-to-use, and possibly easy-to-transfer-to-other-antigen vaccines, as it efficiently outperformed the subcutaneously injected vaccine in the short-term induction of HPV16-neutralizing antibodies [39].

Mannitol was also used in gene editing PEGylated chitosan/CRISOR-Cas9 nucleotide dry powders developed via thin-film freeze drying (TFFD). An increase in nanocomplex size was noticed when the concentration of cryoprotective agents (mannitol, sucrose, and trehalose) was decreased, and a higher concentration of the cryoprotective agent resulted in less aggregation of the nanocomplexes after reconstitution. From SEM images, aggregation to different extents occurred, and a higher concentration of cryoprotective agents resulted in less shrinkage of the disk-like dry powder. Mannitol at 3%, with or without leucine as a dispersion enhancer, was selected as the most suitable for formulation with a preferable aerodynamic performance, despite sucrose and trehalose showing less aggregation and higher transfection efficiency. A trend of increasing nanocomplex size was observed with a decreasing concentration of cryoprotective agents and with the use of the TFFD technique, with no obvious trend observed in terms of the zeta potential of DP formulations. In conclusion, the thin-film freezing technique might be an encouraging preparation method for polymer-based nucleic acid nanocomplex dry powder, followed by lyophilization [40].

### 3.4. Formulation Approaches and Perspectives Using Trehalose as Excipient

Trehalose is another non-reducing disaccharide combined with other excipients, such as moisture protectors (e.g., (tri)leucine). Another thing to consider is safety, because trehalose is not yet included in the inactive ingredient list of the FDA as an excipient for inhalation. The thin-film freeze drying method (TFFD) was used to prepare dry powders of mAb for inhalation. Both trehalose/leucine and lactose/leucine were tested as potential excipients, with lactose/leucine apart from lactose being the only sugar in FDA-approved inhalable products, also presenting excellent aerosol performance. Thus, 1% IgG and lactose/leucine in PBS with anti-PD-1 mAb was chosen. From SEM imaging, TFFD powders were porous with nanoaggregates and low density, corresponding to readily dispersible powders with high FPFs. TFFD technology was applicable in different mAbs, presenting better aerosol performance than shelf freeze drying (shelf FD). Protein loss was observed, in the anti-PD1-1-LL-PBS sample, largely due to binding of the mAbs to surfaces during the TFFD process and due to leucine as the reducing sugar. Thus, more studies need to focus on identifying the proper leucine content in the powder to minimize protein loss and keep the good aerosol properties of the resultant dry powder. Regarding aggregation, powders prepared with mannitol showed the least subvisible aggregates, with the least aggregation in the TFFD formulation prepared with mannitol and without leucine. Thus, excipient type, concentration, properties, vial type, moisture, mAb type, and overall formulation composition can impact the protein recovery, monomer content, and extent of protein aggregation, with clinical research needed to examine the potential advantages of such formulations [41].

Khaled et al. studied aerosolizable dry powders of miR-335-laden induced Extracellular Vesicles (EVs) (iEV-335) generated in B cells for pulmonary delivery via thin-film freeze drying (TFFD). Regarding the morphology, as observed by STEM, the vesicles were polydisperse with no aggregation, with most of them of a suitable size for aerosolization. About >60% of the emitted doses had MMAD values of ≤3 µm, being capable of reaching deep lung regions. From the developed formulations, the trehalose-based (iEV-2) formulation showed the highest Tg; thus, it is preferred to the other stabilizing excipients explored in this study for developing stable thin-film, freeze-dried powders of iEV-335. In conclusion, TFFD technology might be a potential tool for the development of inhalable powder formulations that are effective for the treatment of lung cancer and pulmonary metastasis and are equally effective in downregulating SOX4 gene expression in LM2 human triple-negative mammary cancer cells [42].

In another article, excipient mixtures of trehalose (Tre), dextran (Dex), and shell-forming dispersion-enhancer leucine (Leu) stabilized siRNA-loaded lipid–polymer hybrid nanoparticles (LPNs) during spray drying into nanocomposite microparticles, resulting in inhalable solid dosage forms with high aerosol performance and long-term stability, as observed from a separate study and as mixtures. Preparation of the DPIs was performed by co-spray drying the LPN dispersions with co-dissolved mixtures of Leu and saccharides at various weight ratios. The 40% leucine DPI formulations presented optimal performance in combination with saccharides. Leucine also decreases water adsorption and reduces particle size. DSC thermograms were used to study thermal behavior, with Tg increased when leucine content was higher to Tre-containing DPI. Regarding physical stability, it was present for 6 months, and formulations prepared with combinations of Leu-saccharides maintained higher EE (%). Zeta potential (z-average) values of LPNs increased after spray drying with Dex-Leu/Dex, showing incomplete stabilization, while formulations containing Tre and Leu/Tre presented smaller z-average values after spray drying. Also, DPIs with Leu or combined with saccharides maintained aerosolization performance (yield, Cmax), despite the presence of moisture and application of temperature, during storage. Therefore, a combined use of polysaccharides and Leu is a possible option for a stable DPI [43].

Additionally, an integrated methodology was adopted to accelerate the selection of the best dry powder inhaler excipient system for three model enzymes. Screening using High-Throughput Isothermal Denaturation Fluorimetry (HT-ITDF) was used for three non-reducing sugars and four amino acids for its stabilizing effect on the quaternary structure of the enzymes. All powders displayed, in the selected conditions, 65–85% of FPF, with a noticeable increase in FPF, when a lower L-leucine concentration was combined with trehalose and a higher L-alanine concentration was combined with trehalose. Regarding the kinetic denaturation profiles, all were similar, apart from L-Lysine, with one enzyme (Gox) to exhibit one single plateau and the absence of a lag phase (in the case of SOD). Thus, HT-DSF was found to be a valuable tool to identify the temperature at which HT-ITDF should be performed and the Tout. Also, the HT-ITDF technique was able to spot the most appropriate excipients while all three enzymes maintained their activity and quaternary structures. Thus, with this integrated methodology, a successful narrowing down of 36 potential formulations within hours, with a μg range sample, could be achieved [44].

Solid pharmaceutical forms could also be combined with biopolymers, as has already been observed with PyG, and others such as chitosan or hyaluronic acid with specific characteristics such as low toxicity, biodegradability, and biocompatibility to enhance formulation characteristics and efficiency.

An evaluation and screening of the excipient’s choice and spray solution characteristics on physicochemical behavior and pulmonary suitability of lyospheres, produced via a novel SFD method, was performed. Solutions of different ratios of bulk-forming excipients (mannitol, lactose, poly(vinylpyrrolidone) (PVP), maltodextrin, or hydroxypropyl methylcellulose (HPMC)) and their blends were dispersed. Regarding dry particle size, only mannitol 3% and 1% (*w*/*v*) and lactose 3% (*w*/*v*) resulted in particles within 50–60 μm. Formulations containing mannitol, PVP25, and MDex with at least 1% or mannitol, lactose, PVP25, and MDex at 3% (*w*/*v*) of total solid content in the spray solution were not altered in size during sublimation drying. Also, incorporating more than 70% (*w*/*w*) of a non-crystallizing additional excipient resulted in the formation of amorphous mannitol. Also, the addition of polymeric excipients to a spray solution possibly increased the mechanical stability of lyospheres but also their aggregation tendency. From these findings, mannitol or mannitol/maltodextrin were the best performing formulations, with large porous particles, exhibiting promising performance in the NGI, emitted fractions between 92 and 98% (*w*/*w*) and fine particle fractions of over 55% (*w*/*w*), with a need for a brief characterization of different ratios to ensure quality. Thus, it is important that both the quantity and quality of the excipients have an impact, and a combination of different types of excipients to tailor a suitable formulation is preferred [45].

**Figure 2:**
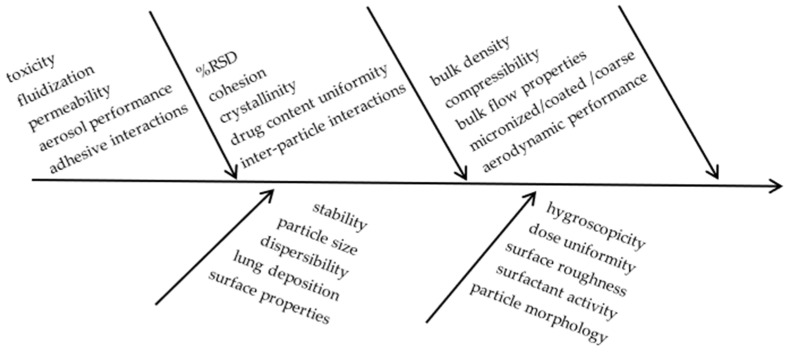
Ishikawa diagram illustrating the different characteristics of the inhalable formulations being affected by excipient selection.

Taking into account all the above literature studies, qualitative and quantitative selection of the excipient combination plays perhaps the most major role in the later characteristics of the inhalable formulation, as was described in the above case studies and as is summarized in the following Ishikawa diagram (Figure 2).

## 4. The Emerging Role of Biopolymers in DPI Formulations

In this part, biopolymers are the dominant functional excipients of each mentioned formulation, in contrast to previously mentioned biopolymers that were used mainly as assisting components.

Extensive research on polymeric particulate systems for pulmonary delivery has been conducted in the last years, as polymers present great advantages such as low toxicity, biodegradability, and biocompatibility, with an unlimited number of applications. Most of the polymers evaluated include natural polymers such as albumin, chitosan (CS), hyaluronic acid (HA), alginates, carrageenan, and gelatin; synthetic polymers such as poly (lactic acid) (PLA), acrylic acid derivatives, poly (vinyl alcohol) (PVA), and cellulose derivatives; and co-polymers such as poly (lactic-co-glycolide acid) (PLGA). Among these polymers, PLGA, HA, and CS are the most promising [7].

Starting off, chitosan is a very useful polymer for pulmonary drug delivery, since chitosan presents mucoadhesive properties which promote drug absorption and increase bioavailability. Thus, the use of chitosan allows for controlled drug release and enhances DPI’s pulmonary deposition. Also, when coupled with thioglycolic acid (TGA), a novel thiomer, a derivative of glycol chitosan (GCS) is generated. However, there are concerns regarding chitosan’s safety and aggregation and surface changes at the physiological pH due to chitosan’s pKa value of 5.5–6.5. This restricts pulmonary delivery and opens a new field of investigation, the chemical modification of chitosan. Hyaluronic acid (HA) is another widely used mucoadhesive polymer. It is naturally present in the lungs, plays a significant role in protecting lung elastin against inflammation, and repairs lung damage. Moreover, it has been reported that HA inhibits phagocytosis in a dose- and molecular-weight-dependent manner. Poly (lactic-co-glycolide acid) (PLGA) is the most studied polymer for pulmonary drug delivery. PLGA is synthetic biopolymer, designed for biomedical use, like natural biopolymers, but is produced through chemical synthesis from lactic and glycolic acids. It presents great advantages since it is a biodegradable and biocompatible polymer. Additionally, it is available in a variety of grades, copolymer compositions, and modified chemical structures. Such variations enable formulators to achieve customized drug release profiles from the formulations, with micro/nano- PLGA carriers having been established. Nevertheless, these might present lung toxicity, polymer accumulation, and an important shift in the microenvironment and pH, as PLGA is characterized by a slow degradation rate (Table 2).

Isoniazid-loaded nanopowders were developed for alveoli targeting with chitosan (CS), thiolated chitosan (TC), and mannosylated chitosan (MC) individually with hyaluronic acid (HA) via both freeze drying and spray drying, which improved powder morphology and flowability. From SEM, mannitol-based powders exhibited a smooth surface and appeared homogeneous, while the surfaces of freeze-dried powders appeared rough due to the remnants of a cross-linker. In a DSC study, freeze-dried nanopowders exhibited no peaks, showing the stability of all components during and after drying, while spray drying with mannitol exhibited an endothermic peak. Also, the % EE of freeze-dried samples was higher, with the drug efficiently loaded after the formation of nanoplexes and strongly embedded in the voids. Drug release was affected by CS modification, which changed its pattern, as controlled release with a longer duration minimized dosing frequency. The ED of the nanopowders was found to be around 90%. It was important that most of the dry nanopowders were deposited in the alveolar region, independently of the drying technique. Cell viability was more than 80%, with mannitol presenting toxicity at high concentrations, optimized in future studies for the safest profile for DPIs. Thus, from both in vitro and in silico pulmonary deposition, mannitol-based nano powders showed better results for future studies [46].

Mannitol was also used in combination with chitosan in a study by Fernández-Paz E. et al. Chitosan (CS)-based nanocapsules (NCs), microencapsulated in mannitol microspheres (Ma MS) via the spray-drying technique, with or without hyaluronic acid (HA), were developed for the treatment of pulmonary diseases. It was observed that HA NCs had a smaller size, inhalable range, and lower positive ζ-potential than those without HA, due to the strong crosslinking effect between HA (negatively charged) and CS (positively charged) on the NC surfaces. Regarding the choice of important parameter values, only the characteristics of morphology, sphericity, and aggregation of MS were selected to be studied, because sizes were not affected by the changing of variables, with all being between 2.0 and 3.7 μm. It was noticed that Ma Ms might act as inert carriers for pulmonary administration, as NCs are released rapidly in an aqueous environment, with no interference from the spray-drying process. Also, from cell studies, high viability was confirmed with high cellular uptake of the nanosystems, maintaining cell integrity. Thus, the presented micro-nano platform for pulmonary delivery seems to be a novel and safe option for future studies [47].

Puerarin was also used to prepare and characterize DPI for pulmonary delivery, reduced to an inhalable size (0.5–2 μm) by hand milling (micronization), and powder mixtures of puerarin, leucine, Mg-St, and lactose prepared by a validated hand-mixing method. From SEM images, it was confirmed that the particle size was of a respirable range, 0.5–2 μm, with homogenous formulations. Using DSC and FTIR, an indication of a possible drug–excipient interaction especially with lactose in the formulation due to hydrogen bonding, was observed [48]. It should be noted that DSC is a reliable method to characterize drug–excipient interactions, cooperativity among excipients, and the crystal/amorphous state of the drug in the mixtures, either in a physical mixture or within carriers during the dosage form development process [49,50]. However, Raman and XRD analysis, useful tools for understanding structural integrity and stability, and HPLC data revealed no interactions. Regarding the aerosolization and dispersion properties, excipients such as lactose, leucine, and Mg-St were considered appropriate excipients for the development of DPI formulations, with the highest FPD (286.3 μg) of puerarin obtained from the formulation containing lactose with the dispersibility enhancer, leucine. Therefore, promising outcomes were presented, such as the FPF of the puerarin DPI formulations of 32–42%, within the range of currently available marketed DPI products, and, thus, the DPI presented here might be used as a treatment of diseases such as diabetes and cardiovascular and neurodegenerative disorders, at a very low dose [48].

Aziz et al. developed a site-targeting montelukast (MTK) sodium microparticle respiratory drug delivery system using spray freeze drying. Screening of the sugars and cyclodextrins as carriers was performed to find suitable excipients for DPI preparation. From the sugars, raffinose displayed the best aerodynamic behavior, with an FPF of 60%, and trehalose presented the lowest. Regarding cyclodextrins, all powders presented acceptable aerodynamic properties, mostly in formulations with α-cyclodextrin, β-cyclodextrin, and highly branched cyclic dextrin (HBCD). As for the physical characteristics (SEM images), highly porous particles in irregular shapes were observed in the sugar-based powder, except for trehalose, resulting in more spherical ones, while CDs presented spherical particles with large porosity. From the XRD diffractograms, amorphous characteristics were indicated, apart from erythritol-containing formulations that complied with a crystal habit. In conclusion, raffinose appears to be the most suitable sugar for good dispersion, aerodynamic characteristics, and solid-state properties (amorphous phase), but the best carriers for MTK seem to be CDs, having a positive impact on overall DPI properties using half the amount of carriers. In particular, α-CDs, with fewer hydroxyl groups, can migrate more to the surface of particles, resulting in less moisture absorption by the particles [51]. However, CDs are related to toxicity and safety issues at higher doses, especially β-CDs, in the context of inclusion complexes. Understanding the toxicological profile of cyclodextrins and their derivatives, as well as their physicochemical properties and mode of complexation, is crucial for ensuring their safe application across different industries [52,53,54]. The best aerodynamic properties (high ED and FPF) are present in formulations containing A-CD, β-CD, and HBCD at 25% and 12.5%. Powders containing mostly CDs showed proper aerodynamic behavior, whereas some formulations showed FPFs higher than 80%, which could be considered an excellent value.

Also, from SEM images of MTK with different sugars, the use of different sugars resulted in differences in particle morphologies. For instance, highly porous particles in irregular shapes were observed in the sugar-based powders. Also, with trehalose as the carrier, particles tended to be more spherical in shape. However, none of the other formulations showed an ideal symmetrical morphology. In addition, some apparent aggregations between irregular particles could influence the flow properties of processed powders [52].

Inhaled protein powder using bovine serum albumin (BSA) as the model protein and 2-hydroxypropyl-beta-cyclodextrin (HP CD) as the protein stabilizer was formulated via the spray–freeze-drying (SFD) technique, and three selected factors for optimization were evaluated in its factorial design: protein content, solute concentration of the feed solution, and atomization gas flow rate. From SEM studies, all formulations presented spherical porous structures, with decreasing particle size when the atomization flow increased and more porous particles when concentration of the solution decreased. It was also noticed that BSA content was a significant factor affecting protein aggregation after SFD, with higher BSA content resulting in a lower level of aggregation [55].

Both are listed in the FDA’s Inactive Ingredient Database. The EMA has approved at least one product (Agilus) using HP-β-CD. However, regulatory approval for pulmonary/inhaled HP-β-CD seems less concrete, remaining in clinical/preclinical studies and not widespread in marketed inhaled products. According to Loftsson et al., in 2005, CDs were considered safe components for pulmonary delivery [56,57]. However, although HP CD was a viable excipient, appreciable aggregation still occurred. Also, to further study the effects of BSA content, extended formulations were designed and prepared, showing very similar aerosol performances, despite BSA content from 0 to 100%. It was observed that aerosol properties were determined by the operating conditions rather than the formulation; thus, different excipients in future work should be included for further optimization [55].

In another formulation, inhalable ciprofloxacin-loaded chitosan sub-micron particles were developed with electrospray as a one-step technique for the treatment of pulmonary infections. The sizes of ciprofloxacin-loaded particles were 386.1 ± 248.5 nm and 501.1 ± 276.3 nm for high- and low-molecular-weight chitosan, respectively, suitable to be used in a dry powder inhaler designed to reach the alveoli. From SEM images, the morphology was spherical, and there was a narrow size distribution. Also, the encapsulation efficiency was above 75%, and a 50% release (first order) in the first 30 min was observed. The low-molecular-weight chitosan had an intrinsic rate that was about 37% higher than chitosan of a high molecular weight, due to fewer interactions between chitosan and ciprofloxacin. It is important to notice that the positive charge of the surface enhances mucoadhesive properties. Also, cytotoxicity assays in A549 human lung epithelial cells showed that ciprofloxacin-containing chitosan particles are safe for use at minimum bactericidal concentration (MBC) doses, and the formulation is biocompatible and effective in a short period of time, retaining its antimicrobial activity against two of the most common respiratory pathogens, *Staphylococcus aureus* and *Pseudomonas aeruginosa* [58].

Additionally, a potential anti-smoking therapeutic in the form of inhaled nicotine hydrogen tartrate-loaded chitosan nanoparticles (NHT-CS) was developed. The formulation was prepared according to a previously published W/O emulsion technique. The locomotor test was used to evaluate the (bio)activity of the formulation in comparison to NHT, indicating that it was bio-active after manufacturing and that biphasic effects of dose response were observed. A Twin-Stage-Impinger (TSI) was used to investigate the in vitro aerosolization and physical characterization of the inhaled nanoparticles. It is noticeable that for 5 min exposure time with a 0.9 L/min flow rate, promising delivery of nicotine was achieved, and accumulated deposition fractions in the pulmonary region were about 19.76% and 21.45% in the tracheobronchial region, while the remaining percent was exhaled. It is important to mention that inhaled NHT-CS might be a viable, non-cancer-inducing, preclinical option for developing novel inhalation formulations and a potential method for better control of nicotine addiction. However, this short-period research does not give certain results about application in exposure studies in C57BL/6 mice and regards long-time use of NHT-CS on lung cells as histopathological change. Additionally, investigations on other pulmonary responses and further pharmacokinetic evaluation, in larger animals, are warranted for a better understanding. The measured deposition in the TSI apparatus suggests potential use for lung targeting, although further testing using aerosol performance methods (e.g., NGI with DPI devices), pharmacokinetic profiling, and long-term pulmonary safety studies is required [59].

Dholakia J. et al. developed a sustained release formulation of genistein (GEN)-loaded, SHMP cross-linked chitosan (CS) nanoparticles for pulmonary delivery with higher stability and anti-diabetic activity. Preparation was performed by the ion gelation method, and nanoparticles were freeze-dried to acquire the powder form. Successful entrapment of GEN with the desirable nanoparticulate range indicated effective entrapment of the crystalline drug via the ion gelation method. The enhanced formulation showed positive z-potential and a particle size of 542.7 ± 136.2 nm, with an observed irregular shape of CS (SEM), in contrast with the crystalline structure of SHMP. Regarding the entrapment efficiency (EE), a higher CS concentration showed an increase in %EE, such as was observed after increasing SHMP, and a dependency on the organic solvent was observed, with greater drug loading when using ethanol. It is important to note that the release was affected by the possible interactions between GEN-CS-SHMP. Additionally, the formulation showed 5-month stability and anti-diabetic activity in the STZ-induced diabetic rat model by promoting insulin sensitization and β-cell proliferation. Therefore, with further studies, this formulation might be a potential next-step treatment for diabetes in the next years [60].

In another approach, spray-dried rifampicin-loaded nanomicellar micro-composites (RFP-NMC) were developed and prepared via an oil-in-water emulsion solvent evaporation technique with stearic acid and chitosan as the hydrophobic and hydrophilic polymers, respectively. From the in vitro dialysis data, 90% was released in 24 h with no toxicity signs. Data was represented with Quality by Design approach software. Also, optimization was performed by the 3^2^ full factorial designs, in which the particle size was depicted as 5.85 µm ± 0.38 µm and the drug content was depicted as 77.20% ± 1.04%. It is noticeable that the characteristic shape of NMCs was useful in increasing both the trajectory and the aerodynamic efficacy, reaching the deeper region of the lungs and leading to better aerosolization and lung deposition. The in vitro lung deposition was evaluated with TSI and ACI. Moreover, with the positive zeta potential (+7.56 mV), the positive charge around the micelles was advantageous for penetrating the alveolar barrier. As a result, there might be increased deposition efficiency and uptake by the macrophages. This formulation, with further studies on enhanced drug delivery, might be a potential therapy for tuberculosis [61].

Moreover, a combination of chitosan–alginate as carrier excipients to formulate a rifampicin DPI was examined, using spray drying as the preparation method. Regarding particle shape, while rifampicin powder presented various nonspherical shapes, a combination of chitosan and alginate in DPI produced rough-surfaced particles (as evidenced by SEM). Also, moisture content was higher in alginate-containing formulations, due to the high viscosity of the alginate solution causing difficulty for water evaporation; thus, more water was entrapped. The aerodynamic properties, such as MMAD, were also different between the combinations, with DPI and with RIF-Ch-Alg 2:1:1 showing the smallest aerodynamic (MMAD) particle size. The entrapment efficiency of DPIs was lower than 50% because of the suspension form of the polymers, with rifampicin not perfectly dissolved and not well entrapped and due to the formation of polyelectrolyte complexes of chitosan and alginate. Drug release was suitable for inhalation only in DPI RIF-Ch-Alg 2:1:1 in both simulated lung fluid and simulated macrophage fluid, containing a phthalate buffer of pH 4.5. The same ratio presented the highest epithelial cell viability (viability 89.73% in concentrations up to 0.1 mg/mL) and possessed an aerodynamic particle size of 11.429 ± 1.259 µm, becoming a potential carrier to develop a dry powder inhaler for tuberculosis therapy and being promising for further studies in vivo [62].

A DPI of vitamin B12 was developed with bovine serum albumin (BSA) as the encapsulating agent, followed by pullulan as a mucoadhesive excipient, resulting in coated micro particles (Vit.B12-Pull-BSA) through spray drying. The particle size of B12-loaded micro-particles was about 590.1 nm, while being respirable. Entrapment efficiency and drug loading presented satisfying results, with 86% API release being recorded at the end of 5 h. Also, high permeability and prolonged residence of vitamin B 12 with an increase in relative bioavailability was observed. Regarding aerodynamic properties, the FPF of drug-loaded micro-particles was 64 ± 6.8%, MMAD was fewer than 5 μm, and GSD was 1.8, reliant on aerodynamic performance. In vitro aerodynamic characterization was performed using no reference to the Twin-Stage Impinger or NGI to be in accordance with USP <601>. From SEM, agglomeration of particles was seen, while MMAD, GSD, and % FPF values showed individual aspiration of micro-particles due to the lack of cohesive forces and superior drug force function. The X-ray diffractogram spectra indicated the amorphous character of the formulation with a low-intensity peak. Thus, in this formulation, the use of natural macromolecule pullulan as a substitute for synthetic polymers improves the mucoadhesion and thermo-protective agent properties of a drug and can be a possible alternative for pulmonary delivery, with an in vivo study illustrating the appropriateness of vit. B 12 containing micro-particles with increased permeability and bioavailability [63].

Also, extra-fine DPIs with chitosan (CS) as a carrier and sodium hyaluronate (SHA) and sodium polyglutamate (SPGA) as polyanions were developed for deep pulmonary delivery of Cyclosporine A (CsA). CS particles were prepared via a modified ionic gelation method by adding the crosslinking agent solution containing CsA to the CS solution. Regarding shape and morphology, drug-loaded DPIs contained spherical particles with wrinkles on the surfaces to reduce interaction between particles and improve the aerosol performance, while those formed by the cross-linking of CS and polyanions had a sponge-like grid structure, which increased drug loading. Also, DPIs were all amorphous, as the absence of a crystal peak indicates, which improved bioavailability. Based on the measured MMAD values, all were extra-fine particles (d < 2 μm). Additionally, the drug release rate of CS-SHA-CsA was faster than that of CS-SPGA-CsA, possibly due to different binding forces of SHA and SPGA with CS. Results from cytotoxicity studies with repeated dose inhalation toxicity indicate that formulations using SHA, SPGA, and CS as carriers are safe and compatible in a drug delivery system. In summary, the formulation showed excellent in vitro aerodynamic performance, non-toxicity, and biocompatibility and could be a future deep pulmonary drug delivery alternative [64].

In another study, a nano-on-microparticle distribution with a physical mixture of solid nanoparticles spread on the surfaces of microscale carrier particles was produced as the model form of a dissolvable small vehicle that carried nanoparticles for deep lung distribution. Chitosan nanoparticles were prepared via the spray-drying method, accompanied by solution atomization and solvent hot air evaporation, while lactose-PEG3000 microparticles prepared with lactose were added to distilled water, with PEG3000 expressed and then spray dried. As for the particle size and shape, it was within the inhalable range and spherical in all cases. Z-potential presented reduction from high-molecular- to low-molecular-weight chitosan, due to the reduced degree of deacetylation. The crystallinity of low-molecular-weight chitosan nanoparticles formulated with a span of 80 and tween 80 exhibited a relatively high level, with profiles of chitosan nanoparticles (adsorbate) having no significant bearing on the fine particle fraction. Chitosan particles physically blended with fine lactose-PEG3000 microparticles exhibited a comparable inhalation performance with commercial dry powder inhaler products. It was concluded that a larger magnitude of zeta potential, higher levels of circularity, and smaller sizes are predicted to give a lower extent of inter-nanoparticulate aggregation and have large interactions with fine lactose-PEG3000 microparticles that aid their delivery to the lower lung regions [65].

## 5. The Case of Minimally Mentioned Nano-Excipients

As was already mentioned, nano-excipients are also present in some of the above-described formulations, mostly as nano-carriers. Nano-carriers are nanosized systems that can range from 1 nm to 1 μm in size. These systems include polymeric nanoparticles, trojan particles, solid lipid nanoparticles, and liposomes. Nanomaterials, especially inhaled nano-micron formulations, have good prospects for application in lung diseases.

The development of DPI formulations of nanoparticles aimed at reducing interparticle attraction and improving DPI performance is considered practical because of the following advantages: (1) formulations are non-invasive and can be delivered directly into the lungs; (2) they exhibit good atomization behavior with desirable stability during processing, administration, and storage; (3) they overcome pulmonary mucus clearance with a reduced incidence of side effects; (4) they minimize the physical instability associated with their liquid state; and (5) most of the drugs delivered in the lungs are hydrophobic, and nanoparticles can also be used to improve the bioavailability of insoluble hydrophobic drugs.

Despite their unique properties, nanoparticles face challenges related to their aerosolization efficiency, potential aggregation leading to poor dispersibility, and intricate engineering requirements for precise dosing. The toxic side effects of bio-nanomaterials must be considered along with the application of these emerging drugs, as well as their short life and instability. A promising approach is nano-in-micro, developed for DPIs to embed the drug within nanosized entities (<1 μm) in microparticles (<5 μm) to take advantage of optimized lung deposition and bypass clearance mechanisms. For instance, inhalable pro liposomal microparticles/nanoparticles of amphotericin B(AmB) with synthetic phospholipids (DPPC, DPPG) and lung surfactant-mimicking phospholipids were designed, developed, and characterized for the treatment of pulmonary fungal infections. It is important to notice that in this study, for the first time, AmB lung surfactant-mimicking micro-nano particles were engineered from advanced organic solution co-spray drying in a closed-mode and high-performance cyclone. The use of phospholipids offers advantages such as biocompatibility, biodegradability, enhanced stability, better solubility, controlled drug release, and appropriate size range for targeted deposition in the respiratory tract. Encapsulation efficiency was also quantified via UV-VIS spectroscopy. All the SD and co-SD powders had a particle size range ≤5 μm, appropriate for inhalation therapy, and were spherical, smooth, and uniform, according to SEM images. Superior aerosol performance (NGI) was achieved [66]. In another study in 2024, liposomal azithromycin DPI was developed for local treatment of chronic respiratory diseases. Preparation using thin-film hydration method was performed, while the average size after spray drying for interaction with bacterial cells tended to be less than 150 nm, with PI less than 0.4, for proper size uniformity in dispersion. The particle size was <105 nm, with a positive surface charge. An improvement in liposome properties after drying was observed when trehalose concentrations increased (17% *w*/*v* formulation giving highest Tg) and when L-leucine was also included. Thus, 0.74% (*w*/*v*) azithromycin-loaded liposomal formulation containing 17% (*w*/*v*) trehalose with 0.5% L-leucine exhibited the best characteristics and was chosen for further study. Acceptable particle sizes and morphology from SEM and a Malvern powder sizer were observed. Regarding kinetics, drug retention over time in an in vitro release setting showed high liposome stability. Also, most interactions were observed after one hour, quicker than previous formulations, with a rapid uptake from bacterial cells and increased activity of azithromycin against *P. aeruginosa* isolates grown in biofilm. Therefore, a rapid and non-cytotoxic delivery to mammalian cells indicates a possible use of this formulation, after further development and studies to improve FPF and limit trehalose use for acceptable stability and higher drug loading [67].

For the first time, the feasibility of inhaled cholesterol–PEG co-modified poly(n-butyl) cyanoacrylate NPs (CLS-PEG NPs) of Docetaxel (DTX) for sustained pulmonary drug delivery in cancer metastasis was tested. NPs were prepared via an emulsion polymerization method. From dynamic light scattering, the particle size of DTX-loaded CLS-PEG NPs was 182.3 ± 3.2 nm with narrow size distribution and a polydispersity index (PDI) of 0.217 ± 0.011. The zeta potential was negative, suggesting that it might prevent aggregation and improve the dispersion stability of NPs. From SEM, smooth to moderately dimpled and raisin-like particles were observed. The FPF of particles prepared by using 10% leucine was found to be 59.44 ± 2.36, indicating better inhalability. From pharmacokinetic data, inhalation formulation prolonged the plasma concentration of DTX for more than 24 h and was quickly and completely absorbed into rat lungs after IT administration. Also, inhalation formulation appeared to bypass the air–blood barrier and dispense to the brain in a sustained release way, presenting non-invasive sustained release. Thus, both freeze-dried and spray-powder CLS-PEG NPs have the potential to be used as a new treatment for the delivery of drugs in the treatment of lung cancer metastasis and brain metastasis [68].

Nanoparticles (NPs), containing a delivery peptide, termed RALA, complexed with plasmid DNA into a dry powder, were developed to determine the parameters of spray drying for an inhalable form. A regular two-factorial design approach was used to generate 19 randomly ordered experiments, combining four parameters and three center points per block, including mannitol concentration, inlet temperature, spray rate, and spray frequency. Most of the particles were in the inhalable range (<5 μm). It was noticed that process yield was increased when mannitol concentration increased, with the mannitol concentration having significant negative effect on DNA recovery, related to the rapid crystallization of mannitol. Regarding z-potential, the positive charge is advantageous for effective cellular uptake. Additionally, from SEM, all samples were dispersible, slightly aggregated, and spherical, with low residual moisture. The results indicated that mannitol concentration was the most important variable affecting all responses, except for encapsulation efficiency. Low mannitol content (1–3%) produced functional RALA/pDNA NPs with the optimal inhalable powder form of RALA NPs, maintaining functionality and desirable powder properties as a potential gene therapy for lung disease treatment, with further studies being needed [69].

In another study, ciprofloxacin (CIP)-loaded poly(2-ethyl-2-oxazoline) (PEtOx) nanoparticles (NPs) for potential pulmonary delivery from dry powder inhaler (DPI) formulations against LRTIs were developed, with NPs prepared with a straightforward co-assembly reaction carried out by intermolecular hydrogen bonding among poly(ethyl oxazoline) (PEtOx), tannic acid (TA), and CIP. NPs presented an inhalable size of 196–350 nm, which increased with higher drug loading. From SEM, CIP-loaded PEtOx NPs showed rough surfaces before and after freeze drying and agglomerates also within the nano range after freeze drying. No interactions were observed between CIP and PEtOx (as confirmed by ATR-FTIR, PXRD, DSC, and TGA analysis). Regarding particle density and FPF, these were increased with higher drug loading, while z-potential decreased. Release studies concluded that drug release was regulated by diffusion and polymer degradation. It was shown that hydrogen bonding between hydrogen donor TA and acceptor PEtOx could be utilized to formulate CIP-loaded PEtOx NPs for lung delivery from DPI formulations. Thus, polymer PEtOx could be a possible carrier for lung drug delivery of the formulation. More studies are certainly needed [70].

Zimmermann et al. developed spray-dried lipid nanoparticles (LNPs), consisting of an ionizable cationic lipid to bind to anionic RNA for efficient encapsulation, as well as a helper lipid, cholesterol, and PEG-DMG encapsulating siRNA. They were based on an updated Onpattro^®^ composition but consisted of a neutral or positively or negatively charged helper lipid (nLNP, (+), (−) LNP) into a successful spray-dried powder at maintained physicochemical properties and siRNA integrity for pulmonary application. It was noticed, from the dual-emission fluorescence spectroscopy method in different excipient solutions (PBS, lactose, mannitol, and trehalose) at different temperatures, that negatively charged (−) LNPs encapsulating negatively charged siRNA might experience repulsion effects, leading them to be less stable than positively (+) charged ones. The best conditions were determined at a 5% lactose solution in combination with a spray-drying inlet temperature of 100 °C. Also, the produced formulation penetrated the lung mucus, maintaining bioactivity with confirmed safety, as well as mediated gene silencing efficiency on mRNA and protein levels both in vitro and ex vivo. As a result, the presented LNPs might be a potential siRNA therapy option for the treatment of asthma, COPD, lung cancer, cystic fibrosis, and viral infections [71].

Also, a proof-of-concept study was conducted with dense poly (ethylene glycol) coated polystyrene nanoparticles (PS-PEG NPs) as model muco-inert particles (MIPs) for powder, using the Excipient Enhanced Growth (EEG) strategy. The MIPs presented improved drug delivery with uniform distribution to the mucosal surface and prevention of drug particles entrapment and elimination by mucociliary clearance. The particle size was 100 nm, smaller than the estimated 500 nm for airway mucus and 150 nm for CF sputum, resulting in effective diffusion. Modified methoxy-PEG-NH2 by the COOH-amine reaction presented a neutral surface charge, and by SEM imaging, discrete spherical particles were observed. Regarding aerodynamic properties, DV50 of the EEG dry powder aerosol was about 2 μm, and the emitted dose was more than 80% of the loading, suitable for pulmonary targeting. PS-PEG NPs presented rapid mucus diffusion in both airway mucus and highly viscoelastic CF sputum by minimizing adhesive interaction. However, when compared to the suspension, diffusion of MIP from the dry powder aerosol was lower, due to the mannitol hygroscopic excipient, which absorbs water and can potentially increase interstitial fluid viscosity. Therefore, dense PEG-coated PS-PEG NPs can be of a micro-meter size formulated via the EEG technique, which could potentially be studied for max MIP loading capacity in EEG powder aerosol formulation and used as a pulmonary disease treatment agent, possibly combined with gene delivery systems in the EEG to improve non-viral gene delivery and ameliorate mucus/sputum drug delivery [72].

Lung-phosphatidylcholine-based lipid nanovesicles (LNVs) of voriconazole for superinfections like aspergillosis were developed via thin-film hydration method preparation and systemically optimized, respecting the QbD principles. Excipient selection—for instance, lung-endogenous phospholipids like DPPC, which are biomimetic to lung surfactants and show no interactions—was performed to reduce air–water interfacial tension, thus preventing lung collapse. HSPC, an aliphatic chain phosphatidylcholine longer than DPPC [73,74], was added as a membrane stabilizer, preventing drug leakage at body temperature, and cholesterol served as a stabilizing and fluidity transmit component to the developed membrane. Moreover, the lung surfactant produced by type II pneumocytes plays a major role in respiratory defense, against infections, and predominantly contains lipids, especially phosphatidylcholines, like dimyristoyl-PC (DMPC) [75]. From FESEM, a polygonal shape of particles with a polydispersity index of 0.154 ± 0.104 and a microdroplet size of ≤5 μm was observed. Regarding drug release, a biphasic pattern with an initial burst release within 2 h followed by a regulated release profile up to 48 h was present. Also, the potential of this formulation was shown from its biocompatibility and safety profile with the pulmonary monolayer and cell lines, along with probable improved drug retention in mouse lungs. Further studies are needed to provide future treatment and prophylaxis for various infections [76].

Dahmash E. et al. produced tyrosine poly-ester-amide nano-loaded nanoparticles with a fixed-dose combination (FDC) of Fluticasone Propionate (FP) and Salmeterol Xinafoate (SAL) via a one-step process of interfacial polymerization, occurring at the interface between the aqueous layer containing tyrosine and the organic layer containing dimethyl malonyl. The average particle size was 21.9 ± 4.5 nm, but the average particle size from laser diffraction was 105.32 ± 20.05 nm. This difference from the two methods could be attributed to the nature of the polymer, which, upon hydration, increased in size during laser diffraction analysis, whereas the TEM revealed the actual size of the NPs. Regarding the negative z-potential, it can aid colloidal de-aggregation of the particles due to repulsive forces between particles. Also, a high emitted dose for both drugs was observed, in addition to an entrapment efficiency of about 90%. From the animal study, the delivery of the formulation to the lower parts of the respiratory system was confirmed. In conclusion, additional research into the possible local action of the formulation in the targeted area for an extended time is needed in order for the formulation to be a cost-effective choice as carrier-based DPI formulation [77].

## 6. Beyond the Categories: A Review of Functionally Diverse, Unclassified Excipients

Nowadays, there is increased research for developing carrier-free formulations. Some FDA-approved DPIs such as Pulmicort^®^ and Bricanyl^®^ have already implemented carrier-free strategies even for low drug dosages such as budesonide and terbutaline, respectively. These new carrier-free formulations provide an attractive alternative for pulmonary drug delivery. In carrier-free formulations, coating is also helpful in preventing cohesion among particles. The non-polar aliphatic amino acid L-leu has been widely investigated to reduce particle aggregation and protect spray-dried powders from moisture [78]. In most cases, the coating is performed by co-spray drying the drug and L-leu, which crystallizes on the particle surface, creating a hydrophobic layer and reducing particle interactions [9,79].

A pure insulin spray-dried DPI product (Ins_SD powder), free of excipients, for post-prandial glucose control was developed and evaluated. The formulation was produced from an acidic aqueous solution of peptides and filled in hard capsules. Because of the low drug powder mass to deliver, both insulin powders were diluted with mannitol, and a comparison with Afrezza powders blended with mannitol was made. It was noticed that pure insulin powder presented a great aerodynamic performance, with an MMAD of 0.9 μm, FPF up to 91.5%, and extra-fine fraction of 76%. A small aerodynamic size results in deep lung deposition of the particles, effective for systemic insulin uptake with a certain risk of particle exhalation. Regarding stability, the main problem with insulin in general, the formulation presented stability for six months, being stored in cold chain and in room temperature on the patient’s hands. Also, the small particle size and absence of excipients in Ins_SD led to the fast dissolution rate of peptide microparticles and, thus, a fast absorption rate. These observations can change daily therapy without the temperature instability problem, achieving great performance and deposition, as Ins_SD/mann administered intratracheally was promptly absorbed, reaching max concentrations 15 min post-dosing, with a rapid decrease in plasma glucose. Also, an excipient-free pulmonary formulation with safety and tolerability will be provided, with fewer regulatory issues [80]. In another study, particle engineering via electro-spraying with montelukast as both an active ingredient and carrier and budesonide was used to develop inhalable DPIs for the treatment of asthma. The excipient-free formulations were composed of a low-dose drug (budesonide) with a high-dose drug (montelukast). Although a high dependency of the particle size from the drug concentration is known, higher concentrations result in larger particles, while increased concentrations can result in unwanted solute crystallization and an unstable electrospray process. From SEM imaging, the electro-sprayed particles were in the respirable range (<5 μm), presenting a spherical, flattened morphology with a smoother surface. Regarding FPF values, it was shown that electro-spraying increased the delivery performance percent of montelukast emission. Possible interactions between budesonide and montelukast and chemical degradation during the particle engineering process were studied via infrared analysis. Also, the formulation with montelukast associated with budesonide, spatially close to each other, resulted in an amorphous structure and an enhanced dissolution rate. As for the anti-inflammatory effect, from the in vitro inhibition of intracellular reactive oxygen species production, it can be concluded that co-delivery of budesonide with montelukast may result in a great therapeutic outcome in asthmatic patients. Therefore, montelukast as a carrier for budesonide both improved aerosolization properties and the dissolution rate but also presented a synergistic pharmacological effect [81].

Marasini et al. studied the effect of using two- (2FN) and three-fluid nozzles (3FN) on inhalable co-formulated dry powders of tobramycin and diclofenac (2:1 and 4:1 *w*/*w* ratios), without excipients. From SEM imaging, particles were heterogeneous and spherical, with corrugated surfaces, with the 2FN formulations at a 2:1 ratio, showing a mixture of smooth and corrugated surfaces. The 2FN formulations were amorphous due to the absence of the characteristic crystalline peaks from both drugs. Regarding particle size, the majority was within the inhalable range (<5 µm), and the 2FN formulations were smaller, while all showed a monomodal distribution. As for the drug release, it significantly depended upon the diclofenac content and the types of nozzles. Diclofenac released slowly and sustainedly from the 3FN formulations (most % of the drug released over 3 h), while increasing the diclofenac content in the 2FN formulation increased its release, and a rapid burst release of tobramycin was observed in the 2FN formulations. The density of the co-spray dried formulations was affected by the nozzle type, with the 2FN formulations presenting lower tapped density, better flowability, and lung deposition. Diclofenac in the 2FN formulations appeared to be evenly distributed with tobramycin in the particles (from low sodium and chlorine counts). Thus, 2FN formulations showed superior aerosolization performance, with a higher FPF for both drugs, likely due to the smaller particle size and the faster burst release rate of drugs and tobramycin. Diclofenac at 2:1 seems to be the preferred formulation [82].

In a different study, Polymeric Lipid Hybrid Nanosized Carriers (PLHNC) for Docetaxel, shRNA, and pDNA were developed to treat multi-drug-resistant lung cancer, combining self-assembly and nano-precipitation approaches. Statistical designs, a Plackett–Burman design (PBD), and a Box–Behnken design (BBD) were used to justify the feasibility and advantages of the QbD approach, with particle size and encapsulation efficiency as quality profile parameters. A sustained release profile was observed with the highest release in pH = 5.5 and with a cationic lipid responsible for holding the negatively charged shRNA and pDNA molecules for a longer period. It was observed that both in vivo and in vitro characteristics were mostly dependent on the PLHNC than on the drug. The QbD prepared PLHNCs were successfully lyophilized using a cryoprotectant (i.e., trehalose and albumin), with step-by-step development. It is important to mention that the presented carrier system offered great advantages in the local pulmonary delivery of the Docetaxel-shRNA plasmid hybrid PLHNCs, with a fine particle fraction of 68.3 ± 2.5%, along with high drug retention (98.3 ± 3.1%), and that the QbD approach might be a useful tool for the development of formulations, in a cost- and time-effective manner. Increased polymer concentration resulted in an increase in the polymer size of the polymeric core. Along with that, increasing the L/P ratio would give additional thickness to the lipid layer over the polymeric core, which is responsible for additional increases in particle size. As for drug input percentage (factor C), it constantly influences the particle size. Nevertheless, more in vitro and in vivo studies about the role of PLHNCs on cancer cells are needed [83].

Additionally, a gelatin capsule in a single-dose dry powder inhaler (DPI) for the treatment of asthma has been studied to evaluate the effect of moisture and temperature, for the first time, on three different products in two different packs, all subjected to 6 months of accelerated and real-time stability tests (ICH guidelines). Respiratory-grade Budesonide, Formoterol, and Tiotropium were developed as DPIs with RS01 inhaler device model 7 (Plastiape, S.p.a., Italy), with a size 3 gelatin capsule, and the flow rate was kept at 69 L per min to generate a pressure drop of 4 kPa in the device by using the empty capsule based on the USP’s recommendations (USP <601> and Ph. Eur. 2.9.18 guidelines for DPI testing). Particles were coated with 1% of Propylene glycol in iso-octane to minimize powder bouncing and grade excipients. In vitro powder deposition was tested. Also, the average delivered dose (using NGI), flow rate (69 L/min to generate a pressure drop), fine particle fraction (FPF), limit of detection (LOD), limit of quantification (LOQ), coefficient of determination (r2), emitted dose (ED), and fine particle dose (FPD) were evaluated, in addition to a stability study (ICH guidelines (Q1R2)). It was observed that all formulations led to a reduction in FPF, but only Formoterol showed a reduction in ED. Finer-grade API and an excipient leads to a higher FPF, and high temperatures and moisture lead to a greater risk of moisture absorption at the initial point. It was also noticed that, in order to achieve the consistency of FPF throughout the shelf life, a judicial combination, validated process, and selection of the right pack are needed [84].

In another study, using Particle Replication in Nonwetting Templates (PRINT), dry powder particles, to improve the efficiency of delivery and minimize the bystander effect of COPD, were developed in two formulations, with uniformity in size and shape. The first, 1 μm in size and containing pollen-shaped particles, contained 35% PRINT-CFI amorphous ribavirin with 55% trehalose and 10% trileucine. The second, a cylinder-shaped crystalline formulation of 0.9–1 μm particles contained 99% PRINT-IP ribavirin and 1% polyvinyl alcohol. As a result, ribavirin-PRINT-IP was well tolerated in healthy and COPD participants after single and repeat dosing and had improved physicochemical properties, with a higher ratio of active drug to excipient per unit dose, which required less powder overall for inhalation (only two capsules). Also, unintended exposure of ribavirin to bystanders was found to be negligible, and systemic concentrations were low following ribavirin-PRINT-CFI administration, thus minimizing the risk of hemolytic anemia, a known side effect of chronic ribavirin administration and teratogenesis. Further clinical research is required to demonstrate the antiviral characteristics and impact of ribavirin-PRINT-IP on COPD viral induced exacerbations and its potential to be utilized at home safely [85].

## 7. Regulatory, Safety, and Real-World Performance Considerations Following DPI Excipients

The increasing clinical importance of dry powder inhalers (DPIs) is clear, with indications of the growing interest in DPI research obtained from a comprehensive search of the clinicaltrials.gov database, revealing a notable rise in clinical studies centered around DPIs. Among these clinical trials, 4 are awaiting participant recruitment, while 15 are actively recruiting. Additionally, 1 study is listed as active but not recruiting, 395 have been successfully concluded, and 15 were terminated [86].

Despite these findings, the regulatory landscape for novel excipients remains a significant hurdle to innovation. Regulatory authorities such as the FDA and EMA still do not evaluate excipients via a dedicated inhalation excipient approval pathway, and, thus, the pool of excipients with established inhalation safety profiles is limited, and the industrial investment in novel materials is consequently slow. While many excipients are considered safe or inactive for the inhaled route, the use of novel excipients necessitates the generation of safety data, which may be obtained from the public domain or proprietary literature or by additional nonclinical (toxicology) testing. However, there is limited guidance on the specific safety and toxicological data required for novel excipients in pulmonary formulations, resulting in an implicit bottleneck for first-in-class materials despite intense research interest in advanced carriers (e.g., nano-aggregates or novel polymers) [87].

Recent toxicology reviews highlight that although many excipients are classified or generally recognized as safe (GRAS), pulmonary toxicity can differ significantly from oral routes. Therefore, both in vitro testing and biological models need to be approved by regulatory authorities to predict respiratory safety. These models must comply with the global trend of reducing animal use in pharmaceutical research. Another challenge, the toxicity of the combination of different excipients in the formulation of DPIs, is narrowly addressed. Furthermore, the toxicity of polymers is unclear due to the risk of accumulation and adverse effects of degradation products [88].

Emerging classes such as cyclodextrins, while promising for improving solubility and aerodynamic behavior in formulations, bring specific challenges because their safety in long-term inhalation use has yet to be fully established, and their application requires careful characterization and justification of dose-dependent effects on the respiratory epithelium. Another gap in the literature on CD-based materials in pulmonary administration is the lack of standardization [89].

Likewise, nano-structured excipients and engineered nanocarriers (e.g., lung surfactant-mimicking phospholipid nanoparticles) show great potential for enhancing deep lung delivery and payload stability. Therefore, cell viability and barrier integrity studies, which demonstrate biocompatibility, should be taken into consideration [90].

In contrast to these emerging materials (Table 3), the comparative performance of excipients in marketed DPI products reflects a longstanding reliance on well-characterized carriers such as α-lactose monohydrate, mannitol, and selected sugars, which provide predictable flow, dispersion, and aerodynamic properties. Lactose remains dominant in many commercial products due to its established safety and acceptable physicochemical properties, despite its limitations, including suboptimal dispersion without fine carriers and a potential Maillard reaction risk with amine APIs, which motivate ongoing interest in alternatives like mannitol or mixed carrier systems. Comparative studies also underline the importance of excipient combinations (e.g., fine lactose with magnesium stearate) and carriers such as cyclodextrins that may deliver enhanced fine particle fraction with improved aerodynamic behavior relative to traditional sugars, but their translation into marketed products remains limited pending further regulatory acceptance and comprehensive safety data. Many of the multifunctional excipients that are discussed in this systematic analysis can be used for the formulation of APIs coming from recent biological research advances [91,92].

## 8. Conclusions and Future Perspectives

**Scheme 1:**
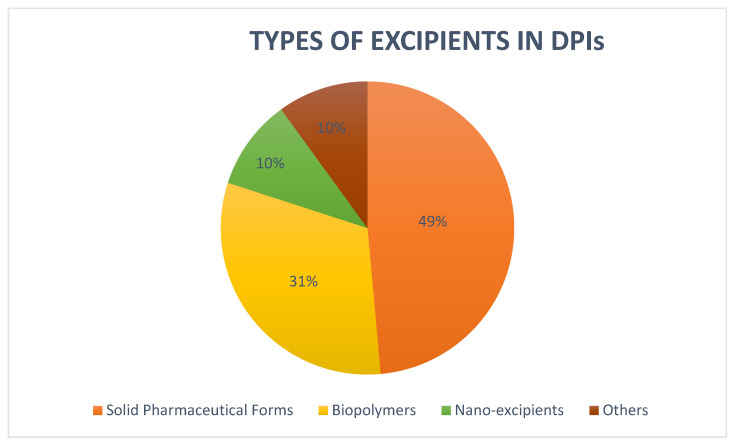
Statistical breakdown of the excipient types in dry powder inhalers from the papers studied.

This review has examined seventy-three articles in detail for recent advances in what is currently known regarding excipients in dry powder inhalers, which are aimed at the treatment of various diseases, mostly pulmonary diseases. From each section, different categories of excipients are highlighted, presenting a holistic view of those, starting from the most common and dominant, present in almost half of the articles—solid pharmaceutical forms, followed by biopolymers at about 30% (Scheme 1). Their extensive use could be attributed to stability, proper aerodynamic properties, and biocompatibility. However, it is important to mention that hygroscopicity, force control, and muco-active behavior present some challenges in their use, due to lung area differences from other drug delivery sites.

**Scheme 2:**
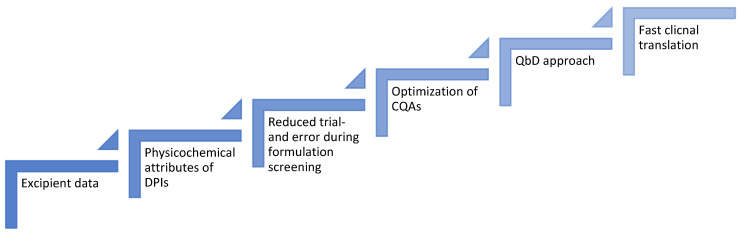
The interconnection between the excipient data and the QbD approach for fast clinical translation of DPIs.

These categories are followed by nano-excipients and others, representing the remaining 20% (Scheme 1, Table 3). Most therapeutic compounds and active pharmaceutical ingredients, mostly anti-asthmatics, anti-inflammatory agents, and antibiotics, have been delivered through coarse lactose carriers with several issues related to using this sugar in respirable dry powders, such as the possible Maillard reaction with amines and the interaction with polar groups. Therefore, drug-loaded formulations have been introduced as carrier-free, some combining a low-dose drug with a high-dose drug and some with only one drug. Another possible option is mannitol, a non-reducing sugar and MgSt combined with lactose. Biopolymers are also an alternative since they present biocompatibility, low toxicity, and biodegradability. Cyclodextrins seem to be a good choice, with great porosity and surface characteristics of the produced particles. On the other hand, nano-formulations do not seem dominant, due to the difficulty in obtaining proper aerodynamic features, such as FPF, and the challenging combination of specific inhalable characteristics with the difficulty of preserving their unique attributes. The development of novel formulations was possible due to the widespread use of modern production techniques, such as spray drying and thin-film freezing. Physicochemical characteristics were also extensively studied, such as particle size and shape, surface morphology, moisture content, zeta potential, flowability, density, and aerodynamic characteristics with fine particle dose (FPD), fine particle fraction (FPF), Mass Median Aerodynamic Diameter (MMAD), and Emitted Dose (ED) being the most mentioned. Different formulations, from common powders, microspheres, sub-micron particles, and nano-formulas to inhalable vaccines, no-carrier formulas, siRNA-loaded lipid–polymer hybrid nanoparticles (LPNs), and nano-embedded formulations with their advantages and shortcomings, as well as their probable future applications, have been discussed in depth. Significant progress in the field of DPI excipients and formulation is expected in the near future due to their interesting properties, the little information known so far, and the variability and unique characteristics they provide for pulmonary drug delivery of different therapeutic compounds. The interconnection between the excipient data and the QbD approach for the fast clinical translation of DPIs is visualized in Scheme 2. In conclusion, the formulation scientist could use this systematic in-depth analysis of the recent literature as a roadmap for the selection of the best excipient in order to formulate APIs with the right drug delivery system. The examples from the recent literature also include the formulation strategy behind the selection of each excipient. This systematic literature review analysis aims to guide future research, regulatory alignment, and smarter excipient selection for next-generation DPIs, especially for biologics or high-potency APIs.

## Figures and Tables

**Table 1:** Diameter of the API in the products, determined by Image-J analyses and scanning electron microscopy (SEM) images of the spray-dried samples. Adapted from [34].

Samples	D ^1^ (nm)	SEM Pictures
NanoMX1_LEU0	134.3 ± 23.07	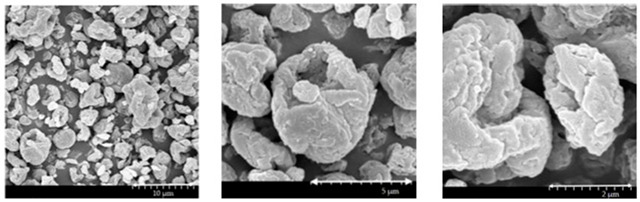
NanoMX1_LEU0.5	126.57 ± 27.26	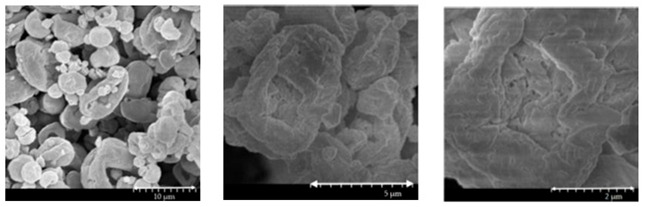
NanoMX1_LEU1	138.27 ± 42.57	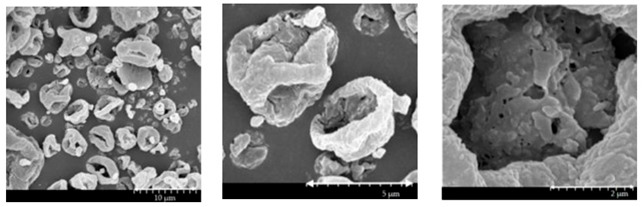

^1^ Data are means ± SD (n = 100 independent measurements).

**Table 2:** Advantages and disadvantages of the utilization of biopolymers as excipients in inhalable formulations.

Biopoylmers	
Advantages	Disadvantages
✓ customized drug release ✓ mucoadhesive properties ✓ absorption enhancers ✓ increasing biavailability ✓ lung elastin protection ✓ lung damage repair	✗ lung toxicity ✗ polymer accumulation ✗ shifts in the microenvironment and pH ✗ slow degradation rate
☞ *Careful study of the biopolymer-excipient effect in physicochemical and aerodymanic properties of the formulation is mandatory*	

**Table 3:** Functional classification and formulation-relevant properties of excipients used in dry powder inhaler formulations.

Excipient Category	Representative Excipients	Primary Role in DPI Formulations	Added Value/Formulation Advantage	Physicochemical Characteristics	Key References
**Excipients as carriers**	a-Lactose monohydrate (InhaLac^®^, Respitose^®^, Pharmatose^®^), Mannitol, Trehalose	Improve powder flow, dose uniformity, and dispersion of micronized API FDA approved	Enable reproducible dosing and scalable carrier-based DPIs; lactose surface modification improves drug detachment and lung deposition; possibility of use of large porous mannitol–leucine (MLAs) microparticles as carriers for deep lung areas	Particle size (micronized vs. coarse), surface roughness, morphology (SEM), bulk density, compressibility, permeability, MMAD	[17,18,19,23,25,38,71]
**Dispersion enhancers/surface modifiers**	L-leucine, Magnesium stearate (MgSt), Fine lactose, Silica microspheres	Reduce inter-particle cohesion; enhance emitted dose (ED) and fine particle fraction (FPF)	Improve aerosolization efficiency, reduce hygroscopicity, and enhance powder fluidization; in drugs with strong drug-lactose adhesion, combination of MgSt and lactose fines can both enhance FPF and reduce the amounts of lactose used; leucine improves spray-drying yield and dispersibility; at higher concentrations, leucine appears to have a dominating influence on the surface of particles	Surface energy, particle morphology (SEM), flowability, cohesion, density, MMAD, FPF	[19,20,21,26,27,28,29,30,31,34]
**Polymeric carriers/nanoparticle matrices**	Chitosan, Tyrosine poly(ester amide) (Tyr-PEA), PEG-PCL, PEGylated chitosan, Poly(vinyl alcohol) (PVA)	Carrier-free or nano-in-micro DPI systems; sustained or enhanced drug release, muco-inert particles (MIPs) for prevention of drug particles entrapment and elimination by mucociliary clearance	Enable nano-in-micro architectures, partial amorphization, mucoadhesive properties absorption enhancers improved dissolution and permeability of poorly soluble drugs; addition of polymeric excipients to a spray solution, possibly increase mechanical stability of lyospheres, and aggregation tendency;	Particle size (nm–µm), morphology (SEM), crystallinity (XRPD, DSC), density, FPF, MMAD, dissolution rate; in vitro aerosolization	[34,45,46,47,58,60,62,64,65,68,72]
**Lipid-based excipients**	DPPC, DSPC, DSPG, DOTAP, DDAB, Cholesterol,	Liposomes/LNPs for pulmonary delivery of small molecules and nucleic acids targeted delivery	Mimic pulmonary surfactant, improve biocompatibility and cellular uptake; enable RNA/mRNA delivery via inhalation	Liposome/LNP size, zeta potential, encapsulation efficiency, morphology (TEM/SEM), FPF, stability; Glass transition temperature (Tg)	[66,67,68,69,71,76]
**Cryoprotectants/stabilizers**	Trehalose, Mannitol, Sucrose, HPβCD	Protect particles during spray drying, TFFD, SFD, or lyophilization	Preserve biological activity of proteins, antibodies, and nucleic acids; reduce collapse, aggregation, and loss of aerosol performance	Glass transition temperature (Tg), morphology (SEM), particle size, crystallinity (DSC/XRPD), residual moisture, density	[37,39,40,41,42,43,52,67,71]
**Cross-linkers/ionic gelation agents**	Sodium hexametaphosphate (SHMP), Sodium hyaluronate (SHA), Sodium polyglutamate (SPGA)	Nanoparticle formation and modulation of drug-release kinetics	Enable tunable release profiles and stabilization of nanoparticulate systems for deep lung deposition; formulations using SHA, SPGA and CS as carriers are safe and compatible in a drug delivery system	Particle size (nm), zeta potential, crosslinking degree, morphology (SEM/TEM), release kinetics	[60,64]
**Protein and biologic stabilizers**	Trehalose, Polysorbate 20, HPβCD	Maintain protein conformation and biological activity	Allow for pulmonary delivery of antibodies and proteins with preserved activity and acceptable aerosol performance	Protein content, integrity (SDS-PAGE), particle morphology (SEM), MMAD, FPF, solubility, residual water content	[29,55]
**Processing solvents and formulation aids**	Methanol, Acetonitrile, Chloroform, DMF, DMSO	Particle engineering, spray drying, polymer synthesis, analytical processing	Enable control of particle size, morphology, encapsulation efficiency, and nano-in-micro formation, pre-formulation improvements	Solvent properties (HPLC grade), solubility parameters, viscosity, polarity, influence on particle morphology	[27,30,77]

## Data Availability

No new data were created or analyzed in this study.

## Abbreviations

The following abbreviations are used in this manuscript:

AD	Aerodynamic Diameter
API	Active Pharmaceutical Ingredient
DPIs	Dry Powder Inhalers
DV50	Median of Particle Volume Distribution
ED	Emitted Dose
EMA	European Medicines Agency
FDA	Food and Drug Association
FEF	Forced Expiratory Flow
FESEM	Field Emission Scanning Electron Microscope
FPF	Fine Particle Fraction
FT-IR	Fourier-Transform Infrared Spectroscopy
HT-ITDF	High Throughput Isothermal Denaturation Fluorimetry
MIC	Minimum Inhibitory Concentration
MMAD	Median aerodynamic diameter
Pe	Peclet number
pMDIs	Pressured Metered Dose Inhalers
RSD	Relative Standard Deviation
SEM	Scanning Electron Microscopy
Tout	Outlet Temperature
XCT	X-ray Computed Tomography
XCT	X-ray Photoelectron Spectroscopy
XRD	X-ray Diffraction
